# Efficacy of biofeedback therapy for chronic constipation in adults: a systematic review and meta-analysis of randomized controlled trials

**DOI:** 10.3389/fmed.2026.1759161

**Published:** 2026-05-28

**Authors:** Yuyuan Tu, Zihao Zhou, Yuanlin Li, Ziyan He, Defu Liao, Zhiren Liu, Boyu Li, Zugang Zhou, Shuangchun Ai

**Affiliations:** 1 School of Health Preservation and Rehabilitation, Chengdu University of Traditional Chinese Medicine, Chengdu, China; 2 Department of Rehabilitation, Mianyang Hospital of Traditional Chinese Medicine, Mianyang, China

**Keywords:** biofeedback therapy, chronic constipation, meta-analysis, randomized controlled trial, safety

## Abstract

**Background and objective:**

Chronic constipation (CC) is one of the most common clinical gastrointestinal illnesses, severely impairing patients' physical function and quality of life. Biofeedback therapy (BFT), a painless and non-invasive therapeutic method, has been applied in the clinical treatment of CC, especially dyssynergic defecation constipation. This study aimed to evaluate the efficacy and safety of BFT for the treatment of CC through a meta-analysis.

**Materials and methods:**

Systematically searched eight databases to identify randomized controlled trials (RCTs) related to CC published from the inception of the database up to August 19, 2025. Methodological quality was assessed using the Cochrane risk of bias tool, and meta-analysis was performed with RevMan 5.4 and Stata 18.0. Evidence quality was evaluated using the GRADE approach.

**Results:**

A total of 3,757 studies were retrieved, and 19 trials were finally selected for inclusion. The results of the forest plot showed that compared with the control group, BFT significantly increased the overall response rate [RR = 1.23, 95% CI = [1.12, 1.34], *P* < 0.00001], weekly defecation frequency [MD = 1.60, 95% CI = [1.37, 1.82], *P* < 0.00001]. Additionally, BFT decreased the constipation symptom score [SMD = −1.07, 95% CI = [−1.36, −0.79], *P* < 0.00001] and quality of life score [SMD = −0.74, 95% CI = [−1.19, −0.29], *P* = 0.001]. However, there was no statistically significant difference in adverse events between the BFT and control groups.

**Conclusion:**

The findings of this study suggest that BFT may have some efficacy in improving symptoms and quality of life in patients with constipation. However, the reliability of the conclusions is limited by the small sample sizes and methodological limitations of the included studies. Future well-designed, large-scale, high-quality RCTs are needed to expand upon these results.

## 1 Introduction

Chronic constipation (CC) is a common gastrointestinal disorder characterized primarily by reduced frequency of bowel movements, incomplete evacuation, straining during defecation, and hard stool consistency, with symptoms persisting for 6 months or longer (1, 2). According to statistics, the global prevalence of CC among adults is approximately 15% (3), and factors such as female gender, advancing age, lower socioeconomic position, and decreased dietary fiber intake may be risk factors for increased CC incidence (4–7). CC has become a global public health concern that severely impairs patients' physical function, mental health, and quality of life, while imposing a substantial burden on healthcare resources (6, 8, 9). Although its pathogenesis is multifactorial, it is primarily related to gastrointestinal motility disorders, pelvic floor muscle dysfunction, disorders of gut-brain interactions, decreased numbers of interstitial cells of Cajal, and dysfunction of the enteric nervous system (10–12). After excluding secondary factors, CC can be further classified into normal transit constipation (NTC), slow transit constipation (STC), and defecation disorder (DD), with dyssynergic defecation being the most prevalent type of DD (1, 13). Current management strategies for CC range from lifestyle modifications to pharmacological interventions, such as laxatives, prokinetic drugs, and prosecretory agents, to treatments such as rectal irrigation and surgical procedures (14). Although these methods may temporarily alleviate symptoms, some patients still experience recurrence or therapeutic failure, and certain treatments may also carry side effects such as bloating, abdominal pain, colonic injury, and adverse cardiovascular events (15–17). Consequently, there is an imperative need for safer, more effective treatment strategies to improve long-term outcomes and quality of life for CC patients.

Biofeedback therapy (BFT), also known as biobehavioral therapy, is an instrument-based intervention that converts information from imperceptible physiological activities into easily recognizable signals. This enables patients to understand the physiological activity status of their pelvic floor muscles and undergo training under professional guidance to rebuild normal defecation reflex pathways, ultimately achieving functional improvement. In addition, BFT is a painless, non-invasive, and safe therapeutic approach that has been applied clinically for CC, particularly suitable for patients exhibiting dyssynergic defecation. A working group convened by the American Neurogastroenterology and Motility Society (ANMS) and the European Society of Neurogastroenterology and Motility (ESNM) has recommended BFT for both short-term and long-term management of constipation with dyssynergic defecation (Grade I, Level A) (7, 18). However, conclusions from previous meta-analyses regarding its efficacy for CC remain inconsistent. Recently, high-quality clinical research in this field has been continuously updated. Therefore, this study aims to conduct a meta-analysis of relevant randomized controlled trials (RCTs) using evidence-based medicine to further evaluate the efficacy and safety of BFT in the treatment of CC.

## 2 Materials and methods

### 2.1 Registration

The protocol for this study has been registered in the International Prospective Register of Systematic Reviews (PROSPERO) under registration number CRD420251135579. It adheres to the Preferred Reporting Items for Systematic Reviews and Meta-Analyses (PRISMA) guidelines (see Supplementary Appendix 1) (19).

### 2.2 Literature search

From the inception of each database up to August 19, 2025, two researchers (TYY and ZZH) independently searched PubMed, the Cochrane Library, Web of Science, Embase, China National Knowledge Infrastructure (CNKI), Sinomed, VIP, and Wanfang Database. Search terms included “constipation,” “defecation,” “defecation disorder,” “biofeedback,” “EMG biofeedback,” and “randomized controlled trial.” Search strategies are detailed in Supplementary Appendix 2. We manually searched the reference lists of all papers and consulted experts to identify relevant studies.

### 2.3 Inclusion and exclusion criteria

The inclusion criteria are outlined below:

1) Participants: Adults diagnosed with CC according to Rome II, Rome III, Rome IV constipation criteria, or other constipation diagnostic criteria (20–22).
2) Interventions: The experimental group received BFT, while the control group received non-biofeedback treatments (such as standard therapy or pharmacological interventions) or sham feedback.
3) Primary Outcome: Overall response rate. Secondary Outcomes: Weekly defecation frequency, constipation symptom score, quality of life score (PAC-QOL), and adverse events. The definition of the overall response rate is the proportion of patients who experience relief or improvement in constipation symptoms. We accepted the specific definitions provided by the studies that contributed data to the quantitative synthesis for this outcome (detailed in Supplementary Appendix 3). Constipation symptom score used different standardized scales or questionnaires.
4) Study Design: Randomized controlled trial.

The exclusion criteria are summarized as follows:

1) Systematic reviews, meta-analyses, animal studies, conference proceedings, dissertations, and duplicate studies.
2) Studies that only reported the results of biofeedback combined with other specific therapies.
3) Studies include patients with secondary constipation (caused by organic diseases, systemic diseases, or medication factors), pediatric constipation, or those with other severe comorbidities.
4) Studies with absent or inaccessible data, full-text access could not be secured after contacting the author.
5) Non-English or non-Chinese language literature.

### 2.4 Study selection

Two researchers (TYY and ZZH) independently screened and extracted data using EndNote X9 software. After removing duplicates, eligible studies were screened according to inclusion and exclusion criteria. The texts of potentially qualifying studies were then reviewed to determine final inclusion. Disagreements were resolved by a third researcher (LYL).

### 2.5 Data extraction

Two researchers (TYY and ZZH) independently extracted the required data from included studies using a pre-designed data extraction form. The primary data extracted comprised: (1) basic information: first author name, publication year, study title, sample size, age, disease duration, intervention duration, and outcomes; (2) baseline characteristics of participants, details of intervention measures (treatment method, feedback modality, treatment frequency, treatment duration, etc.); (3) relevant outcome measures and data. A third researcher (LYL) will be consulted for discussion and decision-making in disagreement.

### 2.6 Risk of bias assessment

Two researchers (TYY and ZZH) independently evaluated the methodological quality of the included studies using the Cochrane Risk of Bias Assessment tool (23). This tool evaluates six domains: selection bias (random sequence generation and allocation concealment), performance bias (participant and personnel blinding), measurement bias (blinding in outcome assessment), attrition bias (completeness of outcome data), reporting bias (selective reporting of study results), and other biases. The tool classifies studies into three risk categories based on their risk of bias: high risk of bias, low risk of bias, or unclear risk of bias. Disputes were settled through consultation with a third researcher (LYL).

### 2.7 Certainty of evidence

The GRADE system categorizes evidence quality into four levels: high, moderate, low, or very low (24). The quality of the study is downgraded due to the following five factors: risk of bias, inconsistency, indirectness, imprecision, and publication bias (25).

### 2.8 Statistical analysis

All statistical analyses were performed using RevMan 5.4 (The Cochrane Collaboration, London, United Kingdom) and Stata 18.0 (StataCorp LLC, College Station, United States). Binary variables were reported as risk ratio (RR), while continuous variables were reported as weighted mean difference (WMD) or standardized mean difference (SMD), with each effect size accompanied by its 95% confidence interval (95% CI) (26). The chi-square test and *I*^2^ statistic were used to assess statistical heterogeneity among studies. A fixed-effects model was employed when *P* > 0.05 and *I*^2^ < 50%; a random-effects model was utilized when *P* < 0.05 and *I*^2^ ≥ 50%. Subgroup analyses were performed based on treatment frequency, feedback modality, and average disease duration. Sensitivity analysis and subgroup analyses were conducted when high heterogeneity was present to ascertain its sources. Furthermore, when more than 10 studies were included, funnel plots and Egger's test were used to assess publication bias (27, 28). Descriptive analysis was used when the data were inappropriate for meta-analysis.

## 3 Results

### 3.1 Selection and inclusion of studies

**Figure 1:**
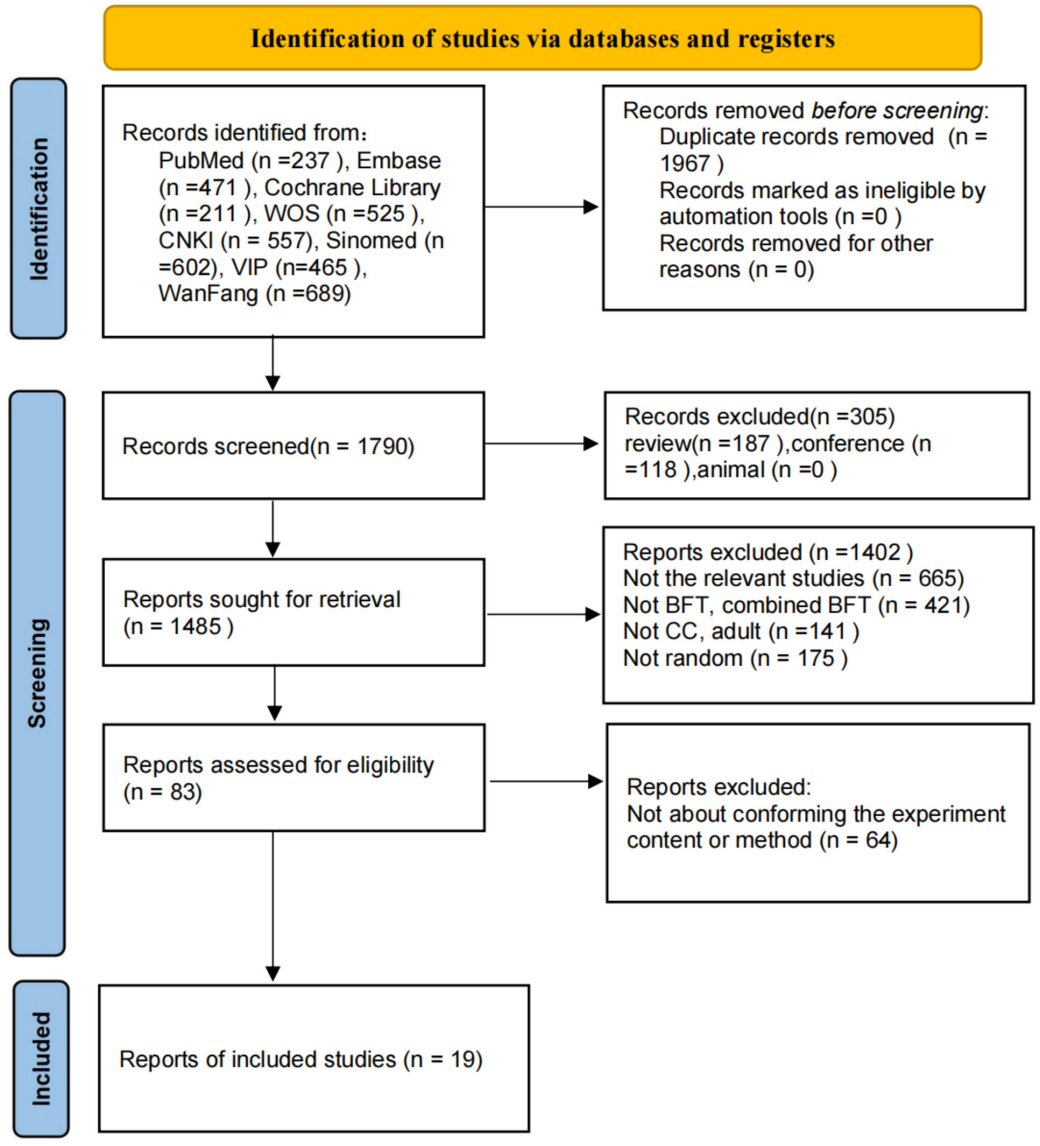
Project for Reporting of Systematic Evaluations and Meta-Analyses (PRISMA) flowchart.

A total of 3,757 studies were initially retrieved from eight databases, distributed as follows: PubMed (*n* = 237), Embase (*n* = 471), Cochrane Library (*n* = 211), Web of Science (*n* = 525), CNKI (*n* = 557), Sinomed (*n* = 602), VIP (*n* = 465), and WanFang (*n* = 689). After eliminating duplicates and a preliminary assessment of titles and abstracts, 3,674 studies were excluded. After a comprehensive evaluation of 83 articles, 19 research studies were ultimately included in the meta-analysis (Figure 1).

### 3.2 Characteristics of included studies

Nineteen studies satisfied our inclusion and exclusion criteria, encompassing 1,288 participants: 646 in the experimental group and 642 in the control group. Participants ranged in age from 20 to 84. Of these studies, 17 (29–45) were two-arm trials, whereas two (46, 47) were three-arm trials. A three-arm study (47) contrasted biofeedback, sham feedback, and standard therapy. The primary areas of the study were China, the United States, Italy, Iran, Turkey, Spain, and Egypt. These 19 research studies utilized different biofeedback protocols, primarily differing in feedback modality, treatment frequency, and course duration. An preliminary analysis of each study's content indicates that eight studies (29, 31, 33, 36, 39, 41, 43, 46) primarily utilized EMG biofeedback, while eight studies (32, 34, 35, 37, 38, 40, 44, 47) mainly employed manometry biofeedback, and three studies (30, 42, 45) could not be determined entirely the feedback modality. Regarding the frequency of biofeedback treatments administered in hospitals, four studies (31, 36, 41, 43) conducted one session per day, one study (42) conducted five sessions per week, ten studies (30, 32–34, 38–40, 44–46) conducted two to three sessions per week, three studies (35, 37, 47) conducted one session per week or less frequently, one study (29) cannot fully determine the treatment frequency. The treatment duration ranged from 30–60 min. Concerning the intervention course, nine studies (31, 33, 34, 36, 39–41, 43, 44) were within 4 weeks, five studies (30, 32, 35, 38, 45) were within 5–8 weeks, three studies (37, 46, 47) were within 3 months, and two studies (29, 42) did not specify course. Interventions for the control group encompassed osmotic laxatives (polyethylene glycol, lactulose), routine therapy (exercise, laxatives, dietary fiber), counseling, and sham feedback, among others. The detailed basic characteristics of the included studies are shown in Table 1.

**Table 1:** Characteristics of included studies.

Study	Sample size (M/F); mean age (years)	Average duration of disease (years)	Interventions		Main feedback modalities	Course of treatment (d/w/m)	Follow-up (w/m/y)	Assessment indicators
			T	C				
Chiarioni et al. (29)	T: 3/51; 33.3 ± 11.12 C: 2/53; 35.1 ± 10.29	NR	EMG-BF: 5 sessions, TT = 30 min	Polyethylene glycol 4,000, 14.6 g orally + 5 counseling, TT = 30 min	EGM	NR	6m, 12m	②
Dai et al. (30)	18/42; 36.38	NR	BF: FREQ = 2 sessions/w, TT = 30–45 min; Simultaneous home training: FREQ = 1–2 sessions/d, TT = 10–15 min	Polyethylene glycol 4,000, 10 g orally, bid	Uncertain	5w	NR	①
Deng and Xiong (31)	T: 31/12; 51.7 ± 9.4 C: 29/14; 52.1 ± 8.7	T: 4.5 ± 2.7 C: 5.2 ± 2.4	BF: FREQ = 1 session/d, TT = 30 min	Lactulose, 15 ml orally, bid	EGM	4w	NR	①
Emad et al. (32)	T: 20/19; 38 C: 19/20; 37	NR	BF: FREQ = 2 sessions/w, TT = NR, 6w	BTX-A injection, 100 units	Manometry	6w	6m	③
Farid et al. (44)	T: 16/8; 39.6 ± 15.9 C: 17/7; 34.7 ± 12.3	T: 4.8 ± 3.34 C: 5.93 ± 3.28	BF: FREQ = 2 sessions/w, TT = 30 min, 8 sessions	BTX-A injection	Manometry	4w	1y	②
Ge et al. (33)	16/30; 34.22 ± 4.89	8.3 ± 6.7	BF: FREQ = 2–3 sessions/w, TT = 50 min	Polyethylene glycol 4,000, 10 g orally, bid	EGM	4w	NR	①
He (34)	T: 16/14; 72.35 ± 5.14 C: 17/13; 72.42 ± 5.23	T: 4.49 ± 1.35 C: 4.51 ± 1.42	BF: FREQ = 2–3 sessions/w, TT = 40–60 min	Polyethylene glycol 4,000 bulk, 10 g orally, bid	Manometry	4w	NR	①
Liu (43)	T: 20/14; 35.6 ± 5.2 C: 22/12; 36.4 ± 4.6	T: 3.4 ± 1.8 C: 3.6 ± 1.7	BF: FREQ = 1 session/d, TT = 30–45 min	The Seton operation method	EGM	4w	NR	①
Nikjooy et al. (46)	T: 4/11; 38.80 ± 13.41 C: 4/11; 38.07 ± 10.41	NR	BF+Standard Treatment: First FREQ = 2 sessions/w, TT = 45 min, 12 sessions; second FREQ = 1 sessions/w, 6 sessions	Standard of Care Treatment	EGM	3m	NR	③
Özkütük et al. (35)	T: 3/9; 40 ± 17 C: 4/8; 38 ± 9	NR	BF: FREQ = 1 session/w, TT=30–45 min	Sham feedback: FREQ = 1 session/w, TT = 30–45 min	Manometry	6w	1w	④
Pan et al. (36)	T: 33/17; 55.6 ± 7.2 C: 33/17; 58.2 ± 6.2	NR	Hospital BF: FREQ = 1 session/d for 20 d, TT = 30 min; post-discharge BF: FREQ = 2–3 sessions/d, TT = 15 min	Lactulose, 15 ml orally, bid	EGM	4w	NR	①③⑤
Rao et al. (47)	T: 3/25; NR C: 2/23; NR	17	BF + standard therapy: FREQ = fortnightly, TT = 60 min, 6 sessions	Sham feedback + standard therapy: FREQ =fortnightly, TT = 60 min, 6 sessions	Manometry	3m	NR	②
Rao et al. (37)	T: 1/12; 48 C: 2/11; 45	NR	BF + standard therapy: FREQ = fortnightly, TT = 60 min, 6 sessions	Standard therapies: exercise, laxatives, dietary fiber, etc.	Manometry	3m	1y	②
Saba et al. (38)	T: 10/11; 40.57 ± 16.97 C: 7/13; 35.20 ± 13.56	T: 6.47 ± 4.67 C: 5.85 ± 4.97	BF: FREQ = 2 sessions/w, TT = NR, 12 sessions	PTNS: FREQ = 3 sessions/w, TT = NR, 18 sessions	Manometry	6w	NR	①④
Shi et al. (45)	T: 25/23; 42.23 ± 9.71 C: 23/21; 40.75 ± 10.50	T: 5.84 ± 2.79 C: 5.45 ± 2.69	Hospital BF: FREQ = 2 sessions/w, TT = 45–60 min; Simultaneous home training: FREQ = 1 session/d, TT = 45 min	Lactulose, 30 ml orally	Uncertain	8w	NR	①③
Si and Zhao (42)	20/50; NR	NR	Hospital BF: FREQ = 5 sessions/w, TT = 30–40 min; Simultaneous home training: FREQ = 1 session/d	Polyethylene glycol 4,000, 10 g orally, bid	Uncertain	at least 10d	NR	①③⑤
Simón et al. (39)	0/20; 75.2	9.3	EMG-BF: FREQ = 2 sessions/w, 4w	Counseling: FREQ = 2 sessions/w, 4w	EGM	4w	3m	②④
Yu et al. (40)	T: 36/34; 70.26 ± 5.24 C: 35/35; 71.29 ± 5.37	T: 6.34 ± 3.28 C: 7.14 ± 3.36	Hospital BF: FREQ = 2–3 sessions/w, TT = 40–60 min; Simultaneous home training: FREQ = 2–3 sessions/d	Polyethylene glycol 4,000, 10 g orally, bid	Manometry	4w	NR	①⑤
Zhang (41)	T: 38/32; 57.5 ± 5.9 C: 38/32; 56.8 ± 6.2	NR	Hospital BF: FREQ = 1 session/d for 20 d, TT = 30 min; post-discharge BF: FREQ = 2 sessions/d, TT = 15 min	Lactulose, 15 ml orally, bid	EGM	4w	NR	①③⑤

M, man; F, woman; T, treatment group; C, control group; w, week; d, day; m, month; y, year; FREQ, frequency; TT, treatment time; qd, once a day; bid, twice a day; tid, three times a day; BF, biofeedback; EGM, electromyography; EMG-BF, Electromyography Biofeedback; CT, conventional treatment; BTX-A, botulinum toxin; PTNS:posterior tibial nerve electrostimulation; PEG, polyethylene glycol; g, gram; mg, milligram; ml, milliliter; ① Overall response rate; ② Weekly defecation frequency; ③ Constipation symptom score; ④ Patient Assessment of Constipation Quality of Life (PAC-QOL); ⑤ Adverse events.

### 3.3 Risk of bias in studies

**Figure 2:**
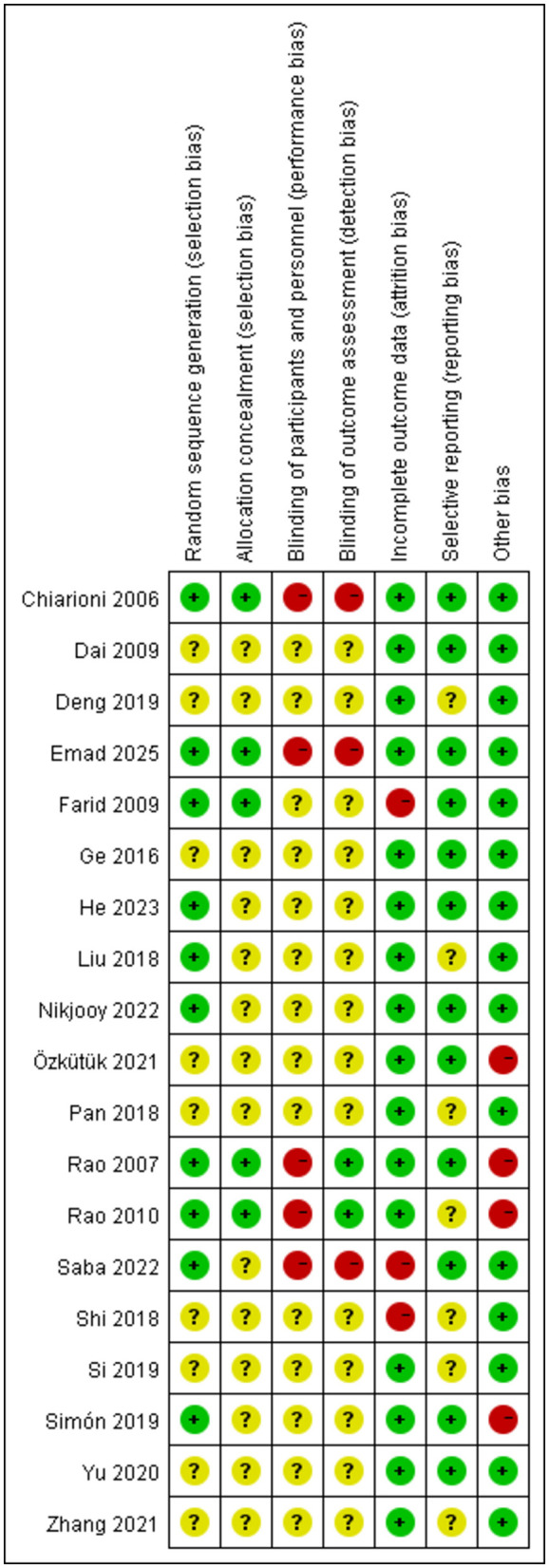
Risk of bias summary.

**Figure 3:**
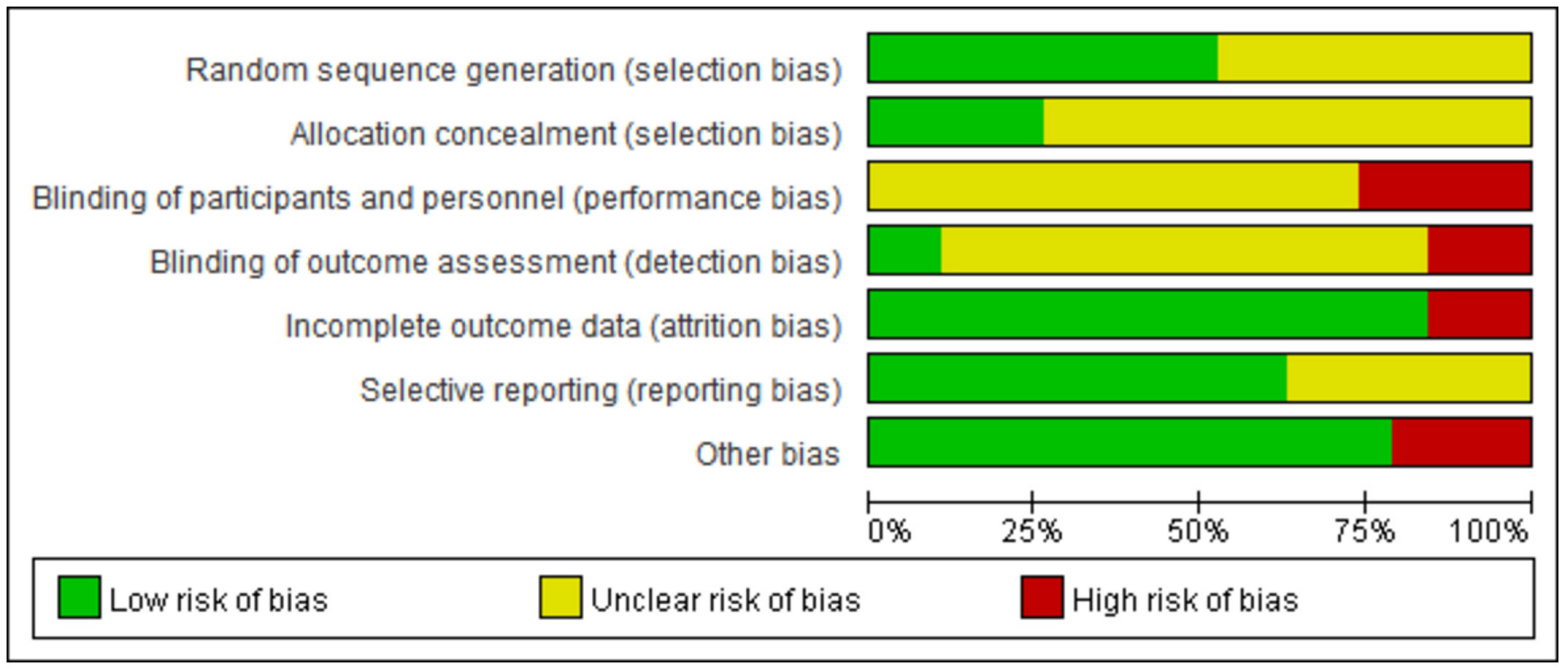
Risk of bias graph.

The risk of bias for each study is shown in Figure 2, and the percentage of risk of bias for each study is shown in Figure 3. All 19 studies used randomization methods; 10 detail the methods used, including random number tables, random sequences, and computer-generated sequences. Five studies describe in detail the use of sealed envelopes for allocation concealment. The remaining studies did not provide details of allocation concealment, so the risk of bias assessment is unclear. Regarding implementation and measurement bias, due to the particularities of BFT, two studies used a partial-blinding design, and the remaining studies did not report blinding procedures. Regarding completeness of outcome data, seven studies reported lost to follow-up or withdrawals, with four adopting the intention-to-treat analysis. Regarding selective reporting bias, seven studies were considered to have some issues potentially. Regarding other biases, 15 studies were classified as low risk, and the remaining four were deemed high risk due to small sample sizes and/or baseline imbalances.

### 3.4 Meta-analysis results

#### 3.4.1 Overall response rate

**Figure 4:**
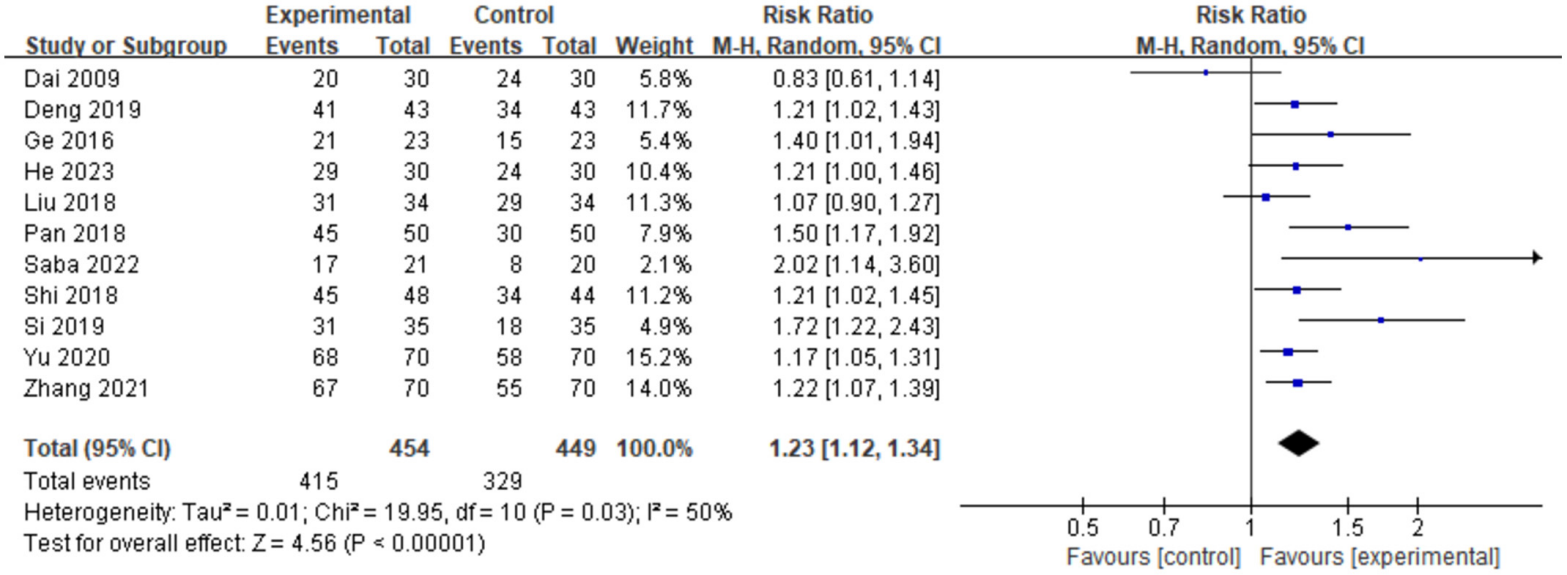
Forest plot of overall response rate.

**Figure 5:**
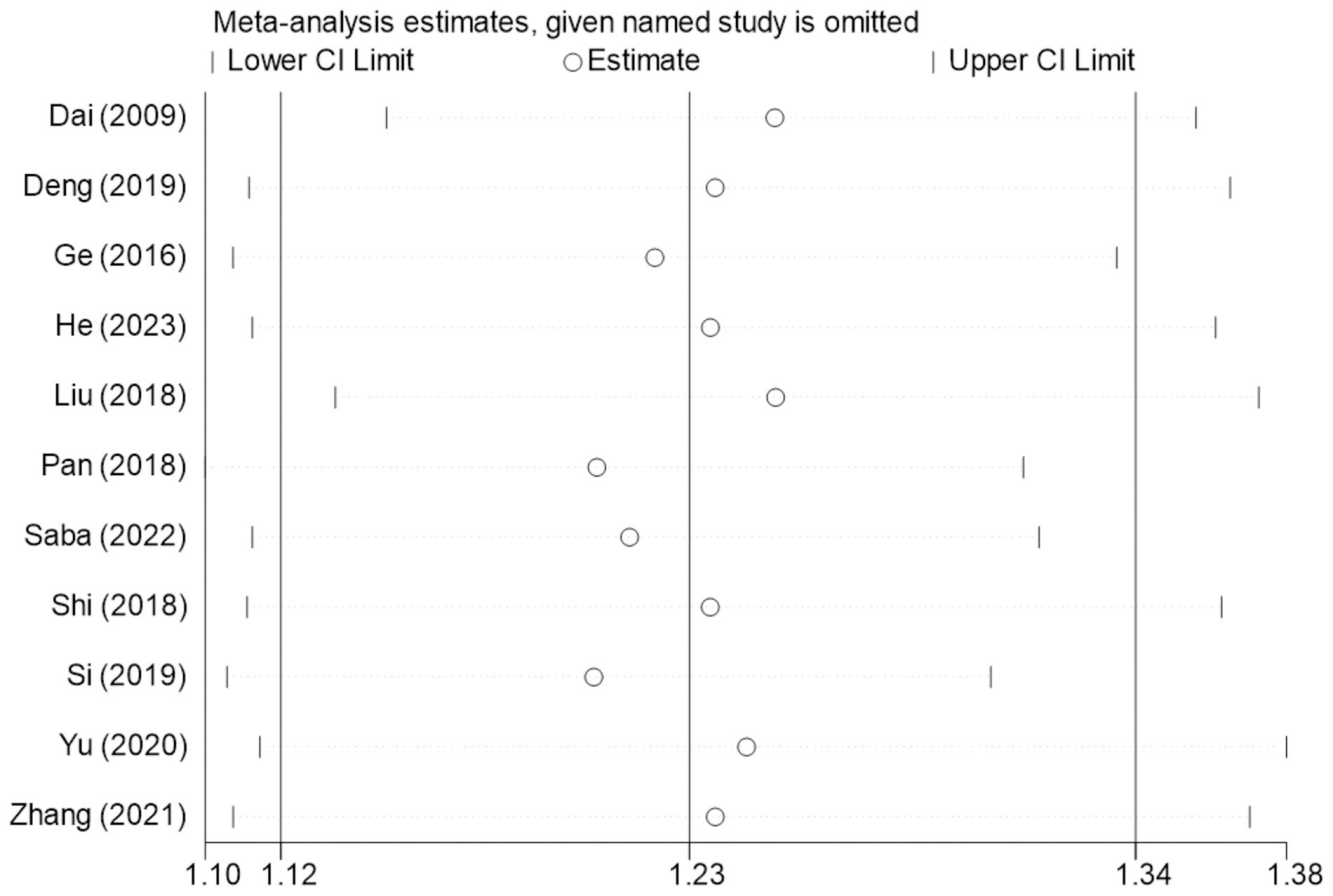
Sensitivity analysis of overall response rate.

A total of 11 studies (30, 31, 33, 34, 36, 38, 40–43, 45) involving 903 patients reported the overall response rate. Due to the moderate heterogeneity among the studies (*P* = 0.03, *I*^2^ = 50%), a random-effects model was used. The results indicated that the BFT group exhibited a superior overall response rate compared to the control group [RR = 1.23, 95% CI = [1.12, 1.34], *P* < 0.00001] (Figure 4). Sensitivity analysis indicated that the results were robust after excluding any of the studies (Figure 5).

In addition, we conducted subgroup analyses to examine the impact of average duration of disease ( ≤ 5 years, >5 years, and unknown), treatment frequency (≥5 sessions/week, 2–3 sessions/week), and feedback modality (EMG, manometry) on BFT efficacy (forest plot in Supplementary Appendix 4). The results of all subgroup analyses were largely consistent with the overall results, suggesting that disease duration, feedback modality, and treatment frequency may not significantly affect the positive effect of BFT on CC.

#### 3.4.2 Weekly defecation frequency

**Figure 6:**
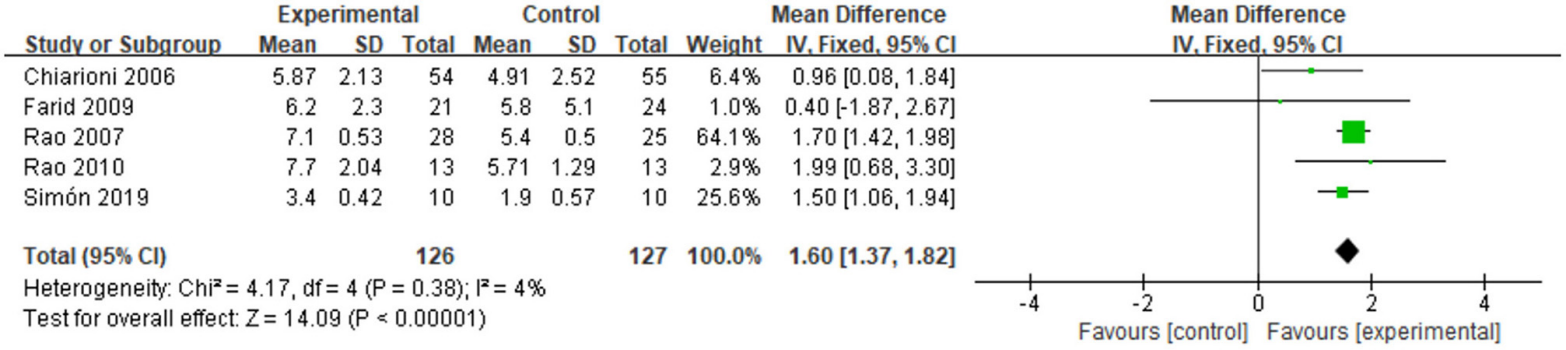
Forest plot of weekly defecation frequency.

A total of five studies (29, 37, 39, 44, 47) involving 253 patients reported the weekly defecation frequency. There was very little heterogeneity among studies (*P* = 0.38, *I*^2^ = 4%), and a fixed-effects model was used. Results indicated that the BFT group demonstrated a superior weekly defecation frequency compared to the control group [MD = 1.60, 95% CI = [1.37, 1.82], *P* < 0.00001] (Figure 6).

#### 3.4.3 Constipation symptom score

**Figure 7:**
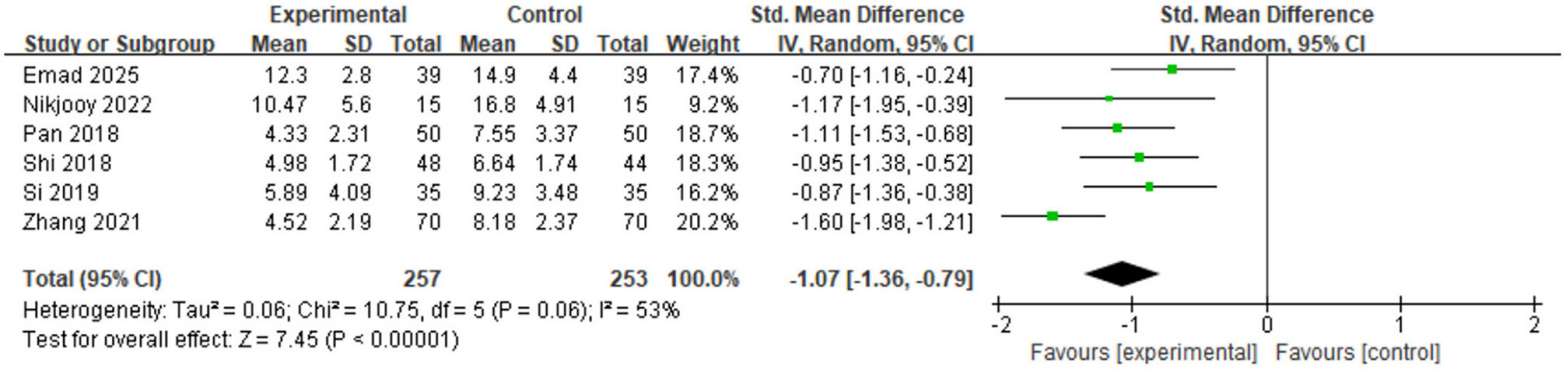
Forest plot of constipation symptom score.

A total of six studies (32, 36, 41, 42, 45, 46) involving 510 patients reported the constipation symptom total score. Due to the moderate heterogeneity among the studies (*P* = 0.06, *I*^2^ = 53%), a random-effects model was applied. Results showed that compared with the control group, BFT reduced the constipation symptom score in CC patients [SMD = −1.07, 95% CI = [-1.36, −0.79], *P* < 0.00001] (Figure 7). Due to the moderate heterogeneity in this study, sensitivity analysis was conducted. After excluding the high-risk research by Zhang et al., the *I*^2^ decreased from 53% to 0%. This discrepancy may stem from the use of a different assessment scale in that study compared to others, thereby contributing to the observed heterogeneity. The results still support that BFT effectively reduced the constipation symptom score in CC patients.

#### 3.4.4 Quality of life score

**Figure 8:**
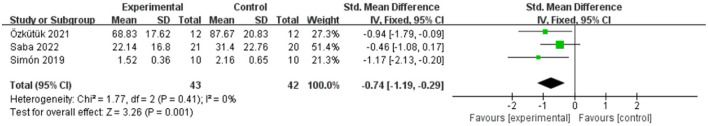
Forest plot of quality of life score.

A total of three studies (35, 38, 39) involving 85 patients reported the quality of life score for patients with constipation. There was no heterogeneity among studies (*P* = 0.41, *I*^2^ = 0%), and a fixed-effects model was applied. Results indicated that compared with the control group, BFT effectively reduced the quality of life score in CC patients [SMD = −0.74, 95% CI = [−1.19, −0.29], *P* = 0.001 < 0.05] (Figure 8).

#### 3.4.5 Adverse events

**Figure 9:**
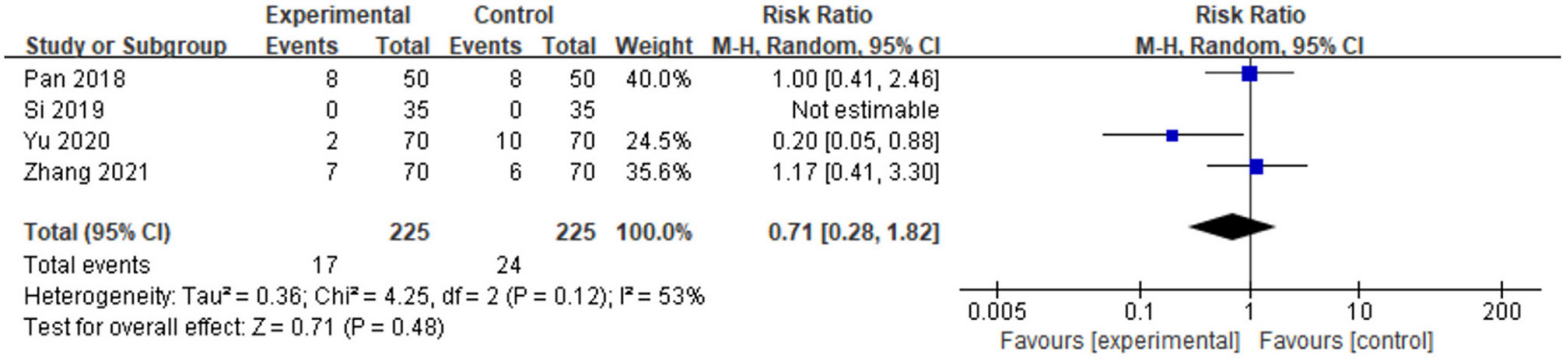
Forest plot of adverse events.

A total of four studies (36, 40–42) involving 450 patients reported adverse events following BFT treatment. One study reported that no adverse reactions occurred in either patient group. The primary adverse events documented included abdominal pain, diarrhea, bloating, hematochezia, and dry stool. There were no adverse events of hematochezia in the experimental group. Due to moderate heterogeneity among studies (*P* = 0.12, *I*^2^ = 53%), a random-effects model was applied. Results showed no statistically significant difference in the overall incidence of adverse events between the BFT group and the control group [RR = 0.71, 95% CI = [0.28, 1.82], *P* = 0.48 > 0.05] (Figure 9).

### 3.5 Risk of publication bias

**Figure 10:**
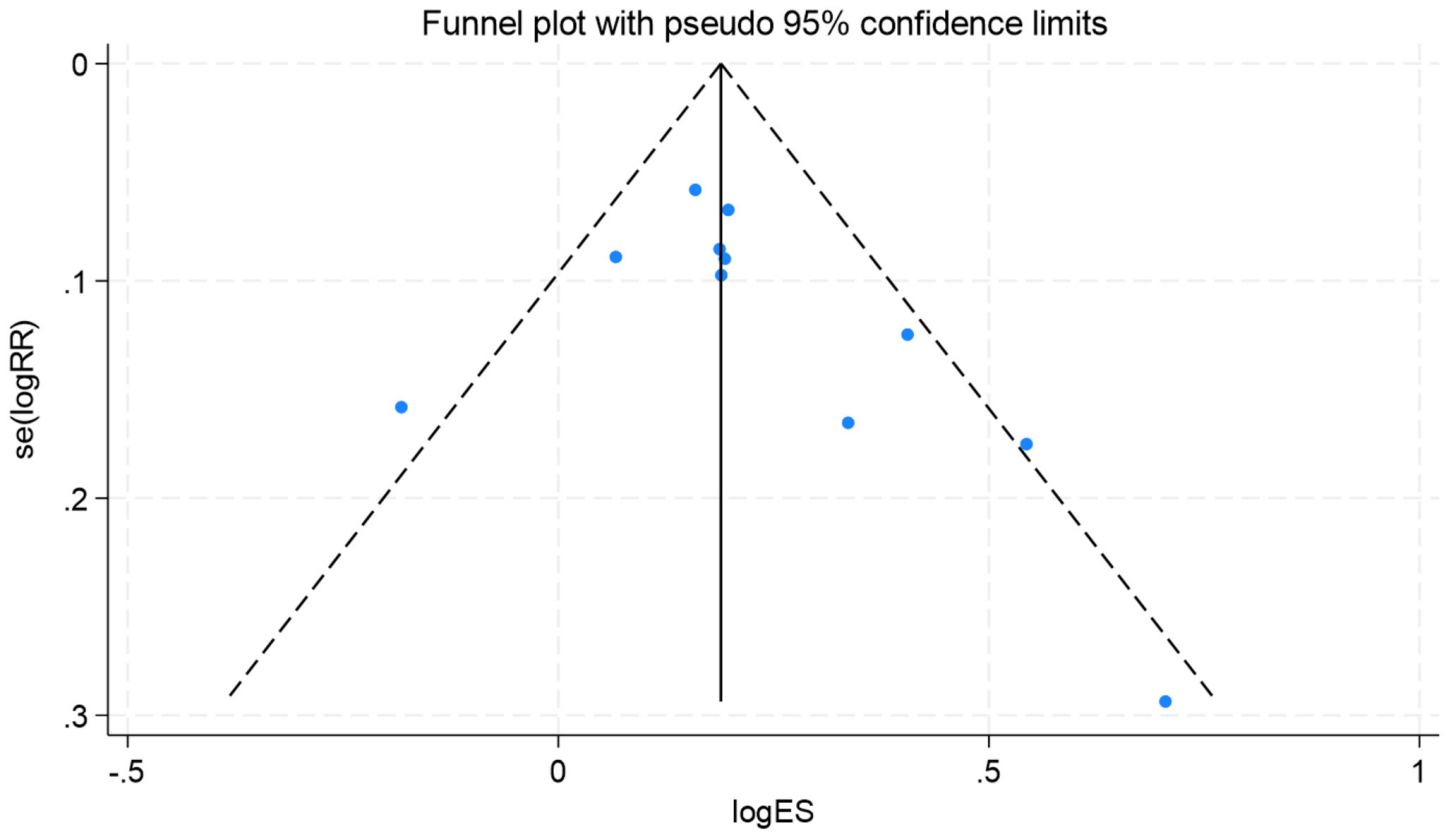
Funnel plot of overall response rate.

As shown in the figure, we detected publication bias using a funnel plot and Egger's test of the overall response rate across 11 study groups. The slight asymmetry on both sides of the funnel plot suggests that there may be potential publication bias (Figure 10), while the Egger's test (*P* = 0.191 > 0.05) indicates no publication bias (see Supplementary Appendix 4).

### 3.6 Certainty assessment

GRADE results are presented in Supplementary Appendix Table 5. The evidence's certainty level was rated as “moderate” for the overall response rate, weekly defecation frequency, constipation symptom score, and quality of life. The certainty of evidence for the adverse events was rated as “very low.” The primary reasons for downgrading were small sample sizes, high heterogeneity, and wide confidence intervals.

## 4 Discussion

### 4.1 Summary of the findings

This meta-analysis included 19 RCTs involving 1,288 patients and systematically evaluated the efficacy and safety of BFT for CC. The results indicated that, compared with control groups, BFT demonstrated considerable improvements in overall response rate and weekly defecation frequency. BFT also reduced the constipation symptom score and quality of life score better than controls. This suggests that BFT may represent an effective therapeutic strategy for improving symptoms and quality of life in patients with constipation. Previous meta-analyses regarding the efficacy of BFT for CC have yielded inconsistent conclusions. A meta-analysis by Patel et al. (48) demonstrated that biofeedback was associated with a significant increase in weekly bowel movement frequency compared to standard treatment. In contrast, Woodward et al. (49) concluded that insufficient evidence exists to draw definitive conclusions about the effectiveness and safety of biofeedback in treating patients with CC. By incorporating more recent, high-quality RCTs, our review provides updated, clearer evidence on the efficacy of BFT.

Furthermore, we conducted subgroup analyses to examine the effects of disease duration, treatment frequency, and feedback modality on overall response rate. The results demonstrated that BFT significantly improved the overall response rate across all evaluated subgroups. Specifically, no statistically significant differences were observed between higher treatment frequency (≥5 sessions/week) and moderate frequency (2–3 sessions/week), nor among different feedback modalities. This suggests that, based on current evidence, no specific treatment frequency or feedback modality demonstrates clear superiority. However, owing to clinical heterogeneity and varied trial designs across the included studies, a formal subgroup analysis of intervention duration was not possible, precluding identification of an optimal therapeutic course. Collectively, these findings imply that the efficacy of BFT may not depend solely on specific intervention details within the scope of this evaluation. Consequently, in clinical practice, clinicians can flexibly tailor prescription elements, such as treatment frequency and duration, to patients' individual situations and therapeutic goals, thereby formulating individualized BFT protocols to optimize therapeutic outcomes. Due to differences in treatment regimens and outcome measures across the studies included in this review, it is not possible to directly assess the long-term efficacy of BFT in patients with constipation. An early small-sample study suggested that the patient's symptoms nearly returned to pre-treatment level after 1 year of follow-up (50). Nevertheless, the results of some subsequent related studies appear to differ from it. Rao et al. (37) found that compared with standard treatment, BFT sustained improvements in symptoms and rectal function among patients with dyssynergic defecation at one-year follow-up; studies by Battaglia et al. (51) and Chiarioni et al. (52) showed that patients with pelvic floor dyssynergia maintained positive treatment effects after 1 or 2 years, whereas those with isolated STC did not. Lee et al. (53) reported that over 80% of initial responders maintained the initial effects during a median follow-up of 44 months. This review includes several studies that reported pooled outcomes for diverse constipation subtypes rather than subtype-specific data, precluding quantitative subgroup analysis by subtype. Nevertheless, the aforementioned evidence indicates that BFT may have an advantage in symptom alleviation and sustained long-term effects for dyssynergic defecation, whereas its benefits for STC remain controversial. Future multi-center, large-sample RCTs are needed to better evaluate the efficacy of BFT in patients with different constipation subtypes.

The primary biofeedback modalities for CC include manometry biofeedback, EMG biofeedback, and sensory biofeedback. Although EMG biofeedback is currently the most widely used clinically, there is insufficient evidence to demonstrate the superiority of any single biofeedback modality. Glia et al. (54) found no difference in efficacy between manometry biofeedback and EMG biofeedback, but this conclusion may be affected by the small sample size. A meta-analysis (55) indicated that the average success rate of studies employing pressure biofeedback was better than that of studies using EMG biofeedback. Conversely, another meta-analysis (56) suggested EMG biofeedback was more effective than non-EMG biofeedback. Recently, there has also been growing interest in comparing the efficacy of biofeedback across treatment settings. Rao et al. (57) reported that home-based biofeedback (HBF) was non-inferior to office-based biofeedback (OBF) for improving intestinal symptoms, pelvic floor muscle physiological function, and quality of life in patients with dyssynergic defecation. Although OBF ensures accurate defecation coordination training through real-time, face-to-face professional guidance, its utility is hampered by frequent hospital visits and higher resource consumption. In contrast, HBF exhibits superior cost-effectiveness and offers the potential to treat more patients in the community, despite the lack of immediate in-person supervision and professional guidance from therapists (58). Therefore, although the studies included in this review are primarily from office settings, HBF appears to be a promising and economical alternative that merits further validation. In addition, we conducted a qualitative analysis of adverse events reported in each study to assess BFT safety, revealing no statistically significant difference between the BFT group and control groups. However, the researchers of the relevant study stated that patients in this group experienced no obvious adverse reactions during BFT. Consequently, BFT is considered a relatively safe treatment with no side effects.

### 4.2 Potential mechanisms of BFT in improving constipation symptoms and bowel function

The mechanism of action of BFT remains incompletely understood. However, clinical studies suggest BFT may improve constipation symptoms through multiple mechanisms. Biofeedback can correct pelvic floor dyssynergia and improve anorectal coordination. In patients with CC, particularly those with pelvic floor dyssynergia, the pelvic floor muscles, such as the external anal sphincter and puborectalis muscle, often exhibit paradoxical contractions during defecation. Through feedback information from biofeedback devices, patients can identify abnormal physiological activity in their pelvic floor muscles and, under appropriate guidance, consciously control and regulate it to restore pelvic floor coordination and reconstruct a normal defecation pattern (18). Concurrently, BFT can also improve the rectal sensory function. For patients with impaired rectal sensation, rectal sensation training through balloon inflation can be employed to adjust the rectal sensory threshold, thereby improving their awareness of defecation and promoting the timely initiation of the defecation reflex (18, 59, 60). Furthermore, research by Emmanuel et al. (61) indicated that BFT for constipation may exert specific effects on the intestines. Laser Doppler measurements showed reduced rectal mucosal blood flow in constipated patients, most pronounced in those with slow-transit constipation. The study further demonstrated a close correlation between rectal mucosal blood flow levels and overall intestinal transit (62). Patients who reported subjective improvement after BFT exhibited significantly increased rectal mucosal blood flow and improved bowel movement frequency. Therefore, the success of biofeedback training may also relate to enhanced autonomic innervation to the intestine and intestinal transit function, thereby increasing bowel movement frequency, reducing dependence on laxatives, and alleviating symptoms such as abdominal bloating.

### 4.3 Strengths and limitations of the study

This study has several advantages over previous meta-analyses on BFT for CC. First, we included the most recent high-quality RCTs and excluded immature and low-quality studies, thereby enhancing the quality of the evidence. A systematic search of Chinese and English literature was conducted to ensure the study's comprehensiveness. Furthermore, we performed a subgroup analysis of overall response rate by key factors, including feedback modality, treatment frequency, and disease duration, to explore the impact of these characteristics on BFT efficacy. Finally, sensitivity analyses, risk of bias assessments, and certainty assessments were conducted to ensure the robustness and credibility of the findings.

This study also has several limitations. First, some included studies reported patient loss to follow-up and withdrawals, reflecting the difficulties of retaining participants in labor-intensive clinical trials such as BFT, which may introduce bias into trial outcomes. Second, all outcome measures in this review are subjective. Due to significant variations in treatment regimens and outcome measures across the included studies, it may not be possible to conduct a meta-analysis of objective indicators such as anorectal manometry or electromyography. Third, differences in biofeedback treatment protocols, such as feedback type, treatment frequency, and duration, along with variations among patient groups, resulted in significant heterogeneity in some findings of this review. Fourth, methodological shortcomings (such as inadequate reporting of random sequence generation, allocation concealment, and blinding implementation), insufficient sample size, and potential risks of bias may compromise the credibility of the study's conclusions. Nevertheless, biofeedback is a form of behavioral therapy, and implementing blinding in behavioral therapy trials presents limitations. Fifth, the concurrent enrollment of diverse constipation subtypes across several studies included in this review, without stratified outcome reporting, precluded subgroup analyses by constipation subtype to precisely assess the differential efficacy of BFT. Therefore, all combined results of this study should be interpreted with caution. Future research should focus on conducting large-sample, multi-center RCTs using validated subjective and objective outcome indicators and standardized biofeedback clinical research protocols. To further verify the short-term and long-term efficacy of BFT for patients with constipation and the efficacy differences of different biofeedback modalities, and optimize treatment regimens to provide more robust evidence-based guidance for clinical practice.

## 5 Conclusion

In summary, BFT may be an effective and safe treatment strategy for improving symptoms and quality of life in patients with constipation, particularly those exhibiting dyssynergic defecation. However, the strength of these conclusions is constrained by the methodological limitations and small sample sizes of the included trials. Future high-quality RCTs are needed to expand on current research and further explore the mechanisms of BFT in patients with constipation.
