# Supplementary material for: Efficacy of biofeedback therapy for chronic constipation in adults: a systematic review and meta-analysis of randomized controlled trials
Source: Front Med (Lausanne). 2026 May 28;13:1759161. doi: 10.3389/fmed.2026.1759161 (PMC13253412; doi:10.3389/fmed.2026.1759161)
Supplement: Supplementary file 2 [file Table_2.docx]

**Search strategies of electronic databases**

| **Database** | **Search strategy** |
| --- | --- |
| Pubmed | ((("Constipation"[Mesh]) OR ((((((((((((((Constipation[Title/Abstract]) OR (Constipated[Title/Abstract])) OR (Constipating[Title/Abstract])) OR (Constipations[Title/Abstract])) OR (functional constipation[Title/Abstract])) OR (achalasia pelvic floor[Title/Abstract])) OR (defecation disorders[Title/Abstract])) OR (dyssynergic defaecation[Title/Abstract])) OR (Dyschezia[Title/Abstract])) OR (Dyssynergia[Title/Abstract])) OR (Dyssynergic[Title/Abstract])) OR (Colonic Inertia[Title/Abstract])) OR (rectal disease[Title/Abstract])) OR (anus disease[Title/Abstract]))) AND (("Biofeedback, Psychology"[Mesh]) OR ((((((((((((biofeedback[Title/Abstract]) OR (biofeedbacks, psychology[Title/Abstract])) OR (psychology biofeedback[Title/Abstract])) OR (psychology biofeedbacks[Title/Abstract])) OR (Biofeedback[Title/Abstract])) OR (Biofeedbacks[Title/Abstract])) OR (biofeedback (psychology[Title/Abstract]))) OR (biofeedbacks (psychology[Title/Abstract]))) OR (feedback, psychophysiologic[Title/Abstract])) OR (feedback, psychophysiological[Title/Abstract])) OR (psychophysiologic feedback[Title/Abstract])) OR (myofeedback[Title/Abstract])))) AND (((("Randomized Controlled Trial" [Publication Type]) OR ("Controlled Clinical Trial" [Publication Type])) OR ("Randomized Controlled Trials as Topic"[Mesh])) OR (((((((((((((((((((Randomized Controlled Trial[Title/Abstract]) OR (Randomized Controlled Trials as Topic[Title/Abstract])) OR (randomized controlled trial[Title/Abstract])) OR (controlled clinical trial[Title/Abstract])) OR (randomized controlled study[Title/Abstract])) OR (randomized trial[Title/Abstract])) OR (randomized study[Title/Abstract])) OR (randomized placebo-controlled study[Title/Abstract])) OR (randomized parallel-group study[Title/Abstract])) OR (randomized placebo controlled[Title/Abstract])) OR (randomized double-blind[Title/Abstract])) OR (Randomized[Title/Abstract])) OR (Placebo[Title/Abstract])) OR (Randomly[Title/Abstract])) OR (Trial[Title/Abstract])) OR (Groups[Title/Abstract])) OR (Clinical Trials[Title/Abstract])) OR (Random[Title/Abstract])) OR (RCT[Title/Abstract]))) |
| Embase | #1 'constipation'/exp  #2 'constipation':ab,ti OR 'constipated':ab,ti OR 'constipating':ab,ti OR 'constipations':ab,ti OR 'functional constipation':ab,ti OR 'achalasia pelvic floor':ab,ti OR 'defecation disorders':ab,ti OR 'dyssynergic defaecation':ab,ti OR 'dyschezia':ab,ti OR 'dyssynergia':ab,ti OR 'dyssynergic':ab,ti OR 'colonic inertia':ab,ti OR 'rectal disease':ab,ti OR 'anus disease':ab,ti  #3 #1 OR #2  #4 'biofeedback'/exp  #5 'biofeedbacks, psychology':ab,ti OR 'psychology biofeedback':ab,ti OR 'psychology biofeedbacks':ab,ti OR 'biofeedback':ab,ti OR 'biofeedbacks':ab,ti OR 'biofeedback (psychology)':ab,ti OR 'biofeedbacks (psychology)':ab,ti OR 'feedback, psychophysiologic':ab,ti OR 'feedback, psychophysiological':ab,ti OR 'psychophysiologic feedback':ab,ti OR 'myofeedback':ab,ti  #6 #4 OR #5  #7 'Randomized Controlled Trial':ab,ti OR 'Randomized Controlled Trials as Topic':ab,ti OR 'randomized controlled trial ':ab,ti OR 'controlled clinical trial':ab,ti OR 'randomized controlled study':ab,ti OR 'randomized trial':ab,ti OR 'randomized study':ab,ti OR 'randomized placebo-controlled study':ab,ti OR 'randomized parallel-group study':ab,ti OR 'randomized placebo controlled':ab,ti OR 'randomized double-blind':ab,ti OR 'Randomized':ab,ti OR 'Placebo':ab,ti OR 'Randomly':ab,ti OR 'Trial':ab,ti OR 'Groups':ab,ti OR 'Clinical Trials':ab,ti OR 'Random':ab,ti OR 'RCT':ab,ti  #8 #3 AND #6 AND #7 |
| Cochrane Library | #1 (Constipation):ab,ti,kw OR (Constipated):ab,ti,kw OR (Constipating):ab,ti,kw OR (Constipations):ab,ti,kw OR (Functional constipation):ab,ti,kw OR (achalasia pelvic floor):ab,ti,kw OR (defecation disorders):ab,ti,kw OR (dyssynergic defaecation):ab,ti,kw OR (Dyschezia):ab,ti,kw OR (Dyssynergia):ab,ti,kw OR (Dyssynergic):ab,ti,kw OR (Colonic Inertia):ab,ti,kw OR (Rectal disease):ab,ti,kw OR (Anus disease):ab,ti,kw  #2 (Biofeedback):ab,ti,kw OR (Biofeedbacks, Psychology):ab,ti,kw OR (Psychology Biofeedback):ab,ti,kw OR (Psychology Biofeedbacks):ab,ti,kw OR (Biofeedback):ab,ti,kw OR (Biofeedbacks):ab,ti,kw OR (Biofeedback (Psychology)):ab,ti,kw OR (Biofeedbacks (Psychology)):ab,ti,kw OR (Feedback, Psychophysiologic):ab,ti,kw OR (Feedback, Psychophysiological):ab,ti,kw OR (Psychophysiologic Feedback):ab,ti,kw OR (Myofeedback):ab,ti,kw  #3 (Randomized Controlled Trial):ab,ti,kw OR (Randomized Controlled Trials as Topic):ab,ti,kw OR (randomized controlled trial):ab,ti,kw OR (controlled clinical trial):ab,ti,kw OR (randomized controlled study):ab,ti,kw OR (randomized trial):ab,ti,kw OR (randomized study):ab,ti,kw OR (randomized placebo-controlled study):ab,ti,kw OR (randomized parallel-group study):ab,ti,kw OR (randomized placebo controlled):ab,ti,kw OR (randomized double-blind):ab,ti,kw OR (Randomized):ab,ti,kw OR (Placebo):ab,ti,kw OR (Randomly):ab,ti,kw OR (Trial):ab,ti,kw OR (Groups):ab,ti,kw OR (Clinical Trials):ab,ti,kw OR (Random):ab,ti,kw OR (RCT):ab,ti,kw  #4 #1 AND #2 AND #3 |
| Web of science | #1 TS=(Constipation OR Constipated OR Constipating OR Constipations OR functional constipation OR achalasia pelvic floor OR defecation disorders OR dyssynergic defaecation OR dyschezia OR Dyssynergia OR Dyssynergic OR Colonic Inertia OR rectal disease OR anus disease)  #2 TS=(biofeedback OR biofeedbacks, psychology OR psychology biofeedback OR psychology biofeedbacks OR Biofeedback OR Biofeedbacks OR biofeedback (psychology) OR biofeedbacks (psychology) OR feedback, psychophysiologic OR feedback, psychophysiological OR psychophysiologic feedback OR myofeedback)  #3 TS=(Randomized Controlled Trial OR Randomized Controlled Trials as Topic OR randomized controlled trial OR controlled clinical trial OR randomized controlled study OR randomized trial OR randomized study OR randomized placebo-controlled study OR randomized parallel-group study OR randomized placebo controlled OR randomized double-blind OR Randomized OR Placebo OR Randomly OR Trial OR Groups OR Clinical Trials OR Random OR RCT)  #4 #1 AND #2 AND #3 |
| CNKI | #1（主题：便秘+功能性便秘+盆底失迟缓综合征+排便障碍+排便失调  (精确)）  #2（主题：生物反馈+生物反馈训练+生物反馈疗法+肌电生物反馈(精确)）  #3 （摘要：随机对照试验+随机对照研究+RCT+随机+对照+控制组+安慰剂+试验+分组+临床+临床研究+临床疗效(精确)） |
| VIP | #1 题名或关键词=便秘+功能性便秘+盆底失迟缓综合征+排便障碍+排便失调  #2题名或关键词= 生物反馈+生物反馈训练+生物反馈疗法+肌电生物反馈  #3 摘要=随机对照试验+随机对照研究+RCT+随机+对照+控制组+安慰剂+试验+分组+临床+临床研究+临床疗效 |
| Sinomed | #1 ("便秘"[常用字段:智能] OR "功能性便秘"[常用字段:智能] OR "盆底失迟缓综合征"[常用字段:智能] OR "排便障碍"[常用字段:智能] OR "排便失调"[常用字段:智能]) OR ("便秘"[不加权:扩展])  #2 ("生物反馈"[常用字段:智能] OR "生物反馈疗法"[常用字段:智能] OR "生物反馈训练"[常用字段:智能] OR "肌电生物反馈"[常用字段:智能]) OR ("生物反馈, 心理学"[不加权:扩展])  #3 ("随机对照试验"[常用字段:智能] OR "随机对照研究"[常用字段:智能] OR "RCT"[常用字段:智能] OR "随机"[常用字段:智能] OR "对照"[常用字段:智能] OR "控制组"[常用字段:智能] OR "安慰剂"[常用字段:智能] OR "试验"[常用字段:智能] OR "分组"[常用字段:智能] OR "临床"[常用字段:智能] OR "临床研究"[常用字段:智能] OR "临床疗效"[常用字段:智能]) OR ("随机对照试验"[不加权:扩展]) |
| Wangfang | #1 主题=便秘 OR 功能性便秘 OR 盆底失迟缓综合征 OR 排便障碍 OR 排便失调  #2 主题=生物反馈 OR 生物反馈训练 OR 生物反馈疗法 OR 肌电生物反馈  #3 主题=随机对照试验 OR 随机对照研究 OR RCT OR 随机 OR 对照 OR 控制组 OR 安慰剂 OR 试验 OR 分组 OR 临床 OR 临床研究 OR 临床疗效 |
