# Supplementary material for: Efficacy of biofeedback therapy for chronic constipation in adults: a systematic review and meta-analysis of randomized controlled trials
Source: Front Med (Lausanne). 2026 May 28;13:1759161. doi: 10.3389/fmed.2026.1759161 (PMC13253412; doi:10.3389/fmed.2026.1759161)
Supplement: Supplementary file 3 [file Table_3.docx]

**Summary Table of Overall Response Rate Evaluation Criteria**

| **Study** | **Criteria for the overall response rate evaluation** |
| --- | --- |
| Dai et al. 2009 | Criteria for Assessing Clinical Efficacy: Before and after treatment, scores were assigned for the following items: frequency of bowel movements, stool consistency, Bristol stool type, defecation difficulty, and incomplete evacuation. Each item was scored on a scale of 0–3. Criteria for determining efficacy: (1) Clinical cure: Symptoms resolved, with a total score of 0. (2) Marked improvement: Symptoms significantly improved; total score reduced by ≥2/3 compared to pre-treatment. (3) Improvement: Symptoms improved; total score reduced by ≥1/2 compared to pre-treatment. (4) No improvement: No improvement in symptoms; total score unchanged or reduced by <1/2. Overall response rate = (Clinical cure + Marked improvement + Improvement) / Total number of cases × 100% |
| Deng and Xiong 2019 | The criteria for assessing clinical improvement are categorized as significant, moderate, fair, and poor. Overall response rate = (Number of significant cases + Number of moderate cases + Number of fair cases) / Total number of cases × 100% |
| Ge et al. 2016 | Criteria for Assessing Clinical Efficacy: Before and after treatment, scores are assigned for abdominal distension, abdominal pain, straining during bowel movements, incomplete evacuation, and a sensation of anal obstruction. A score of 3 indicates severe symptoms that interfere with daily life and work; a score of 2 indicates moderate discomfort with no impact on daily life or work; a score of 1 indicates mild symptoms; and a score of 0 indicates no symptoms. The total clinical symptom score was calculated. Patients whose symptoms completely disappeared after treatment were considered cured; those whose total score decreased by ≥75% with significant symptom improvement were considered markedly improved; those whose total score decreased by ≥50% with slight symptom improvement were considered effective; and those whose total score decreased by <50% with no symptom improvement or worsening were considered ineffective.Overall response rate = (Cured + Markedly improved + Effective) / Total number of cases × 100% |
| He 2023 | a. Marked improvement: Symptom relief, smooth bowel movements, return to normal stool consistency, and spontaneous bowel movements within 2 days; b. Improvement: Symptom relief, generally smooth bowel movements, soft stools, and spontaneous bowel movements >3 times per week; c. No improvement: No improvement in condition or even worsening. Overall response rate = (Number of cases with marked improvement + Number of cases with improvement) / Total number of cases × 100% |
| Liu 2018 | Cured: The patient exhibits no clinical symptoms such as decreased bowel movement frequency, prolonged defecation time, hard stools, or difficulty defecating, and bowel movements are smooth; Effective: Clinical symptoms are significantly alleviated, and bowel movements are essentially normal without the need for assistance; Ineffective: Clinical symptoms show no significant improvement, and defecation remains difficult. Overall efficacy rate = (Cured + Effective) / Total number of cases × 100% |
| Pan et al. 2018 | Clinical efficacy is classified into four categories: cured, marked improvement, improvement, and no improvement. “Cured” indicates that the patient’s clinical symptoms have disappeared and no recurrence was observed at the 6-week follow-up; the score is 0. “Marked improvement” indicates that the patient’s clinical symptoms have improved significantly, with a score reduced by more than two-thirds compared to pre-treatment levels, and no recurrence was observed at the 6-week follow-up. “Improvement” indicates that the patient’s clinical symptoms have improved to some extent, with a score reduced by more than half compared to pre-treatment levels. "No improvement" indicates that the patient’s clinical symptoms have not improved, and the total score has not decreased compared to pre-treatment levels. Overall response rate = (Cured + Marked improvement + Improvement) / Total number of cases × 100% |
| Saba et al.2022 | The primary outcome measure was assessed using the Modified Obstructed Defecation Score (MODS)  (i) improvement: if the patient had at least 50% improvement in the outcome measure after therapy. (ii) No improvement: if the patient had less than 50% improvement in the outcome measure after therapy |
| Shi et al.2018 | The scoring system, developed in accordance with the Rome III criteria, consists of five indicators, each scored on a scale of 0 to 5 points.  Efficacy rate (%) = [(Total symptom score before treatment – Total symptom score after treatment) / Total symptom score before treatment] × 100%. An efficacy rate of ≥75% is considered "significant"; ≥50% and <75% is "fair"; ≥25% and <50% is "moderate"; and <25% is "poor." Overall response rate (%) = [(Number of cases with significant efficacy + Number of cases with fair efficacy + Number of cases with moderate efficacy) / Total number of cases] × 100% |
| Si and Zhao 2019 | Constipation was assessed using the Wexner Constipation Scale.  Efficacy Index = (Pre-treatment score – Post-treatment score) / Pre-treatment score × 100%. Marked improvement: efficacy index ≥ 60%; Improvement: efficacy index 20%–60%; No improvement: efficacy index < 20%. Overall response rate = (Marked improvement + Improvement) / Total number of cases × 100%. |
| Yu et al. 2020 | Cured: The patient’s stool consistency is normal; they can pass stool spontaneously once within 2 days; defecation is effortless; there is no sensation of incomplete evacuation; and related clinical symptoms have disappeared. Markedly improvement: Stools are noticeably softer; the patient passes stool spontaneously more than 3 times per week; defecation is effortless; and related clinical symptoms have largely disappeared. Effective: Spontaneous bowel movements no more than twice weekly, stools are hard and dry, occasional use of laxatives is required, and related clinical symptoms show some improvement; Ineffective: Constipation and related clinical symptoms show no improvement or have worsened. Overall response rate = (Cured + Marked improvement + Effective) / Total number of cases × 100% |
| Zhang 2021 | The criteria for evaluating treatment efficacy are categorized as complete recovery, marked improvement, improvement, and no improvement. Complete recovery: The patient’s constipation symptoms have completely disappeared; Marked improvement: The patient’s constipation symptoms have significantly improved; Improvement: The patient’s constipation symptoms have improved to some extent; No improvement: The patient’s constipation symptoms have not improved and may even have worsened. Overall response rate = (Complete recovery + Marked improvement + Improvement) / Total number of cases in this group × 100 |
