# Supplementary material for: Efficacy of biofeedback therapy for chronic constipation in adults: a systematic review and meta-analysis of randomized controlled trials
Source: Front Med (Lausanne). 2026 May 28;13:1759161. doi: 10.3389/fmed.2026.1759161 (PMC13253412; doi:10.3389/fmed.2026.1759161)

**1 Subgroup Analysis Forest Plot of Overall Response Rate**

**1.1 Treatment frequency**

**1.1.1 Fixed effects model**

**
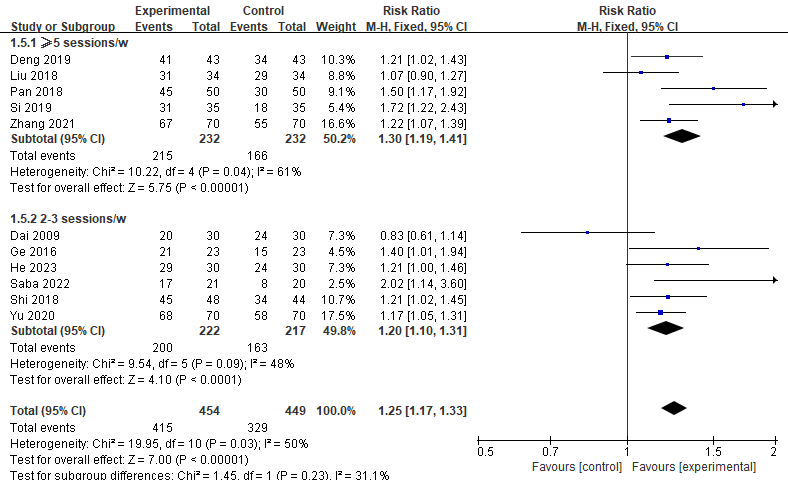
**

**1.1.2 Random effects model**

**
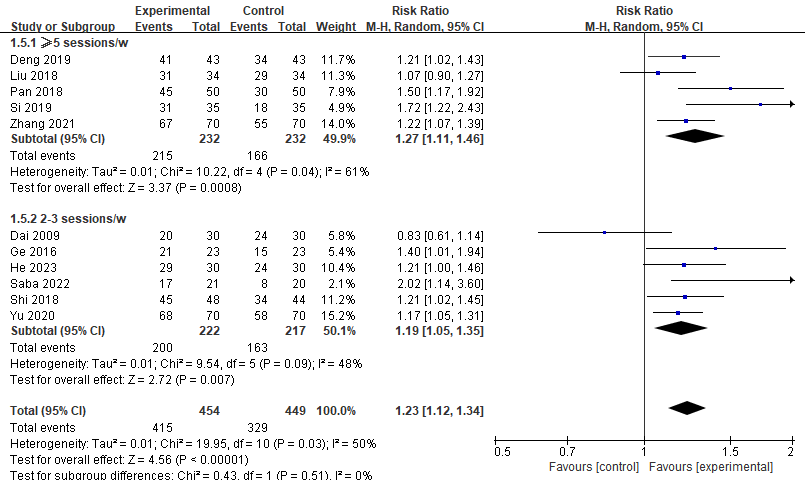
**

**1.2 Feedback Modality**

**1.2.1 Fixed effects model**


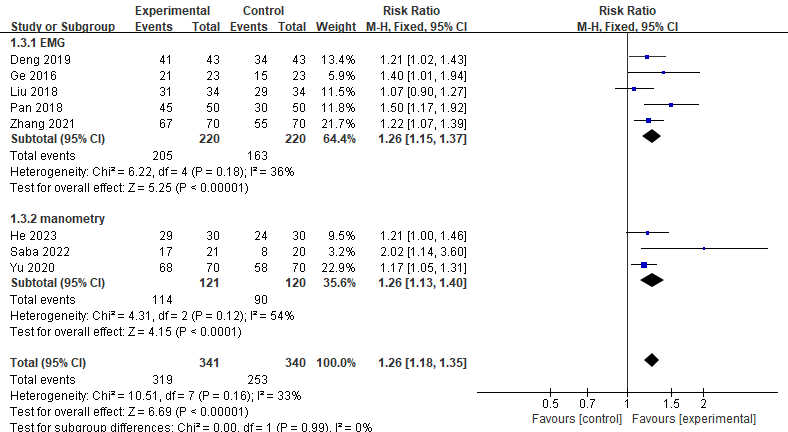


**1.2.2 Random effects model**


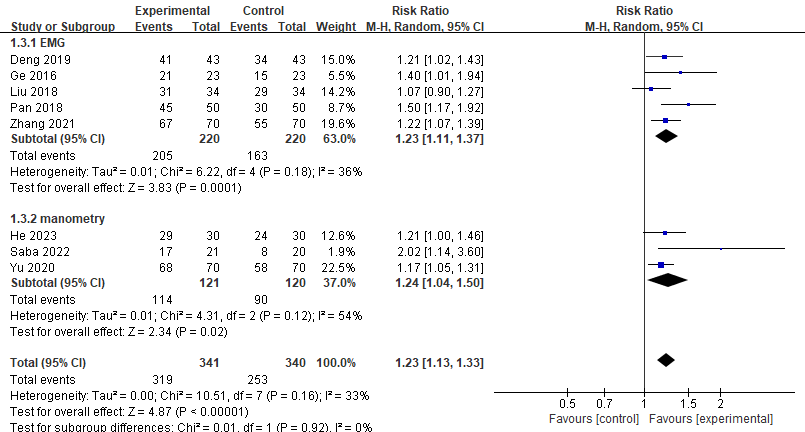


**1.3 Average duration of disease**

**1.3.1 Fixed effects model**


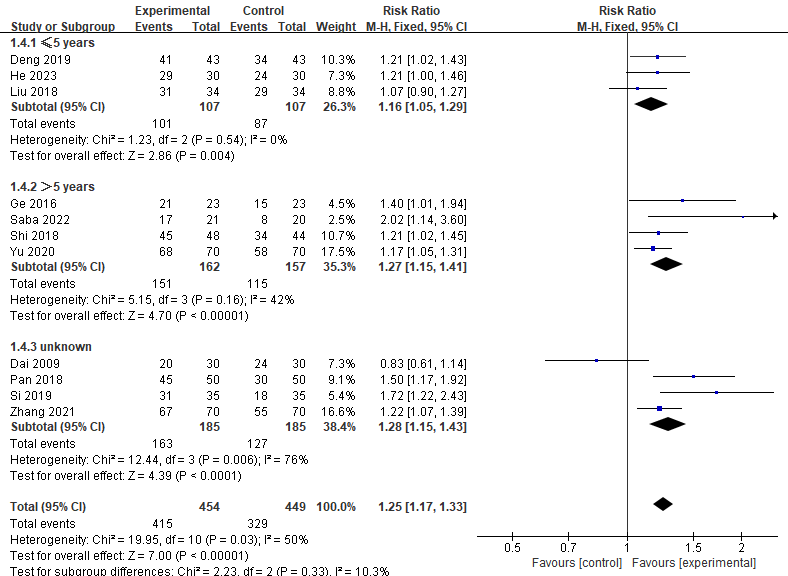


**1.3.2 Random effects model**


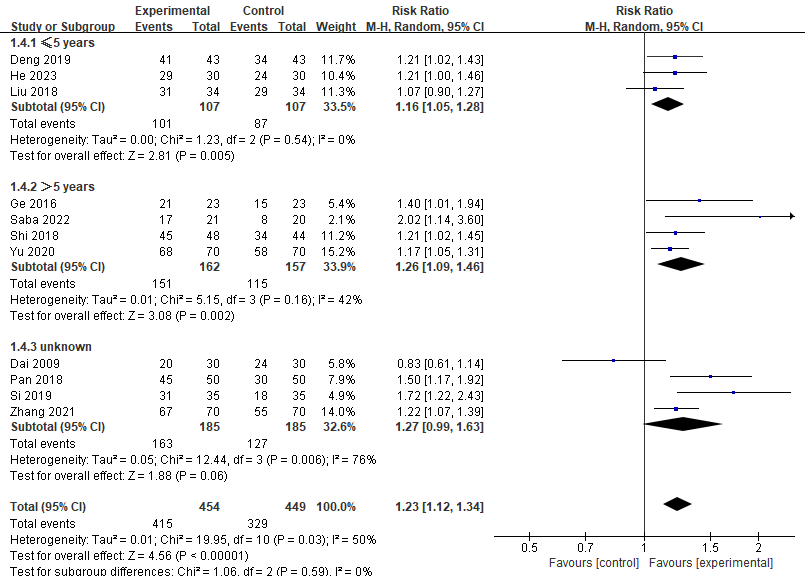


**2 Publication bias test**


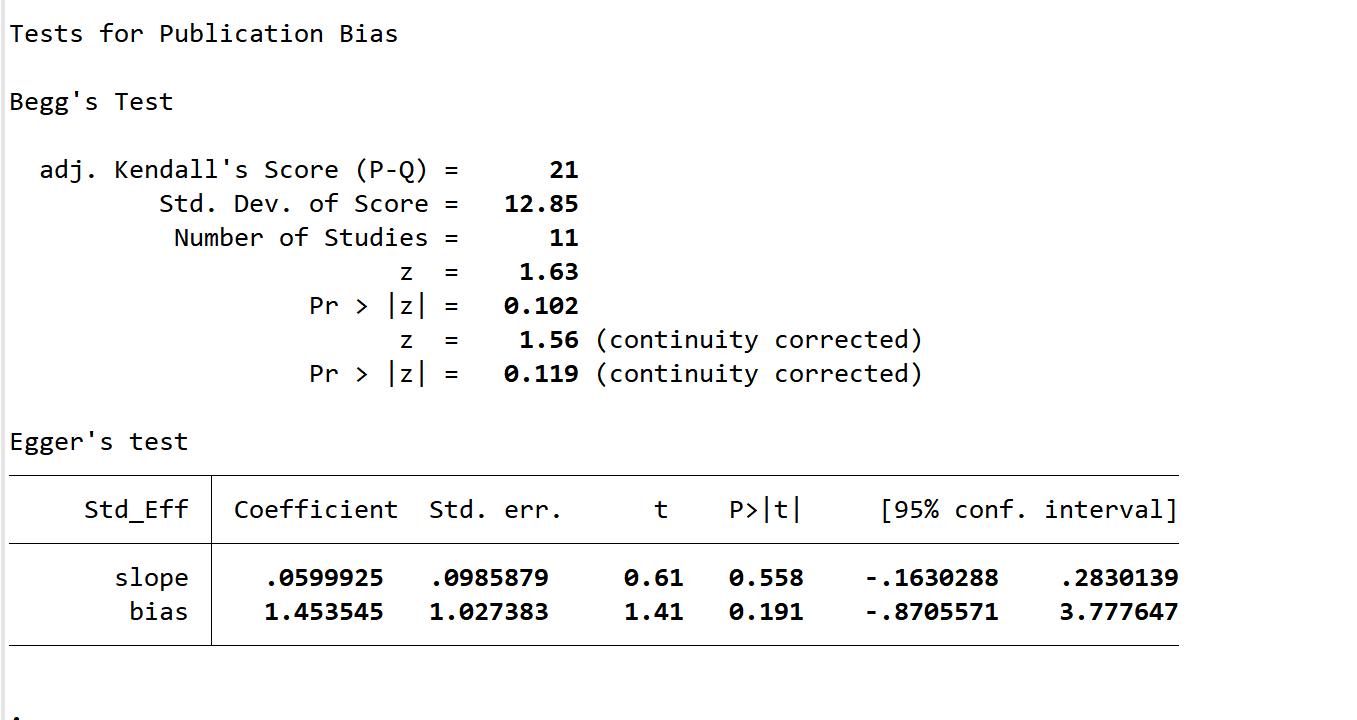

Supplement: Supplementary file 4 [file Table_4.docx]
