# Supplementary material for: Efficacy of biofeedback therapy for chronic constipation in adults: a systematic review and meta-analysis of randomized controlled trials
Source: Front Med (Lausanne). 2026 May 28;13:1759161. doi: 10.3389/fmed.2026.1759161 (PMC13253412; doi:10.3389/fmed.2026.1759161)
Supplement: Supplementary file 5 [file Table_5.docx]

**Author(s): Yuyuan Tu**
**Date:** 2025-11-05
**Question:** Should BFT be used for constipation?
**Settings:**
**Bibliography:** Cochrane Database of Systematic Reviews [Year], Issue [Issue].

| **Quality assessment** | | | | | | | **No of patients** | | **Effect** | | **Quality** | **Importance** |  |
| --- | --- | --- | --- | --- | --- | --- | --- | --- | --- | --- | --- | --- | --- |
|  |  |  |  |  |  |  |  |  |  |  |  |  |  |
| **No of studies** | **Design** | **Risk of bias** | **Inconsistency** | **Indirectness** | **Imprecision** | **Other considerations** | **Experimental** | **Control** | **Relative (95% CI)** | **Absolute** |  |  |  |
| **Overall response rate** | | | | | | | | | | | | |  |
| 11 | randomised trials | no serious risk of bias | serious^1^ | no serious indirectness | no serious imprecision | none | 415/454 (91.4%) | 329/449 (73.3%) | RR 1.23 (1.12 to 1.34) | 169 more per 1000 (from 88 more to 249 more) | ⊕⊕⊕O MODERATE | CRITICAL |  |
| **Constipation symptom score (Better indicated by lower values)** | | | | | | | | | | | | |  |
| 6 | randomised trials | no serious risk of bias | serious^1^ | no serious indirectness | no serious imprecision | none | 257 | 253 | - | SMD 1.07 lower (1.36 to 0.79 lower) | ⊕⊕⊕O MODERATE | CRITICAL |  |
| **Weekly stool frequency** | | | | | | | | | | | | |  |
| 5 | randomised trials | no serious risk of bias | no serious inconsistency | no serious indirectness | no serious imprecision | reporting bias^2^ | 126 | 127 | - | MD 1.6 higher (1.37 to 1.82 higher) | ⊕⊕⊕O MODERATE | CRITICAL |  |
| **Quality of life (Better indicated by lower values)** | | | | | | | | | | | | |  |
| 3 | randomised trials | no serious risk of bias | no serious inconsistency | no serious indirectness | no serious imprecision | reporting bias^2^ | 43 | 42 | - | SMD 0.74 lower (1.19 to 0.29 lower) | ⊕⊕⊕O MODERATE | IMPORTANT |  |
| **Adverse events** | | | | | | | | | | | | |  |
| 4 | randomised trials | no serious risk of bias | serious^1^ | no serious indirectness | very serious^3,4^ | none | 17/225 (7.6%) | 24/225 (10.7%) | RR 0.71 (0.28 to 1.82) | 31 fewer per 1000 (from 77 fewer to 87 more) | ⊕OOO VERY LOW | IMPORTANT |  |

^1^ 50% ≤ I^2^ ＜75%
^2^ The sample size of the included studies was too small.
^3^ RR < 0.75 or > 1.25
^4^ 95% CI contains 1
